# Matching science to reality: how to deploy a participant-driven digital brain health platform

**DOI:** 10.3389/frdem.2023.1135451

**Published:** 2023-05-05

**Authors:** Ileana De Anda-Duran, Phillip H. Hwang, Zachary Thomas Popp, Spencer Low, Huitong Ding, Salman Rahman, Akwaugo Igwe, Vijaya B. Kolachalama, Honghuang Lin, Rhoda Au

**Affiliations:** 1 Department of Epidemiology, Tulane University School of Public Health and Tropical Medicine, New Orleans, LA, United States; 2 Department of Epidemiology, Boston University School of Public Health, Boston, MA, United States; 3 Department of Anatomy and Neurobiology, Boston University Chobanian & Avedisian School of Medicine, Boston, MA, United States; 4 Framingham Heart Study, Boston University Chobanian & Avedisian School of Medicine, Boston, MA, United States; 5 Boston University Alzheimer’s Disease Research Center, Boston University Chobanian & Avedisian School of Medicine, Boston, MA, United States; 6 Department of Medicine, Boston University Chobanian & Avedisian School of Medicine, Boston, MA, United States; 7 Department of Computer Science and Faculty of Computing & Data Sciences, Boston University, Boston, MA, United States; 8 Department of Medicine, University of Massachusetts Chan Medical School, Worcester, MA, United States; 9 Department of Neurology, Boston University Chobanian & Avedisian School of Medicine, Boston, MA, United States

**Keywords:** digital phenotyping, Alzheimer’s Disease, technology, study design, feasibility

## Abstract

**Introduction:**

Advances in digital technologies for health research enable opportunities for digital phenotyping of individuals in research and clinical settings. Beyond providing opportunities for advanced data analytics with data science and machine learning approaches, digital technologies offer solutions to several of the existing barriers in research practice that have resulted in biased samples.

**Methods:**

A participant-driven, precision brain health monitoring digital platform has been introduced to two longitudinal cohort studies, the Boston University Alzheimer’s Disease Research Center (BU ADRC) and the Bogalusa Heart Study (BHS). The platform was developed with prioritization of digital data in native format, multiple OS, validity of derived metrics, feasibility and usability. A platform including nine remote technologies and three staff-guided digital assessments has been introduced in the BU ADRC population, including a multimodal smartphone application also introduced to the BHS population. Participants select which technologies they would like to use and can manipulate their personal platform and schedule over time.

**Results:**

Participants from the BU ADRC are using an average of 5.9 technologies to date, providing strong evidence for the usability of numerous digital technologies in older adult populations. Broad phenotyping of both cohorts is ongoing, with the collection of data spanning cognitive testing, sleep, physical activity, speech, motor activity, cardiovascular health, mood, gait, balance, and more. Several challenges in digital phenotyping implementation in the BU ADRC and the BHS have arisen, and the protocol has been revised and optimized to minimize participant burden while sustaining participant contact and support.

**Discussion:**

The importance of digital data in its native format, near real-time data access, passive participant engagement, and availability of technologies across OS has been supported by the pattern of participant technology use and adherence across cohorts. The precision brain health monitoring platform will be iteratively adjusted and improved over time. The pragmatic study design enables multimodal digital phenotyping of distinct clinically characterized cohorts in both rural and urban U.S. settings.

## 1. Introduction

Longitudinal research paradigms generally involve in-clinic methodologies administered at various time points for the duration of the study period (Andersson et al., 2019). The ongoing pandemic caused an unprecedented transformation, which led to the implementation of telehealth and remote testing that exposed gaps in traditional research frameworks (Neumann et al., 2021), the utility of remote assessment to increase participation, and the potential of technology to improve the feasibility of observational studies. Internet-connected applications and devices can enable a patient-driven focus that, combined with data-driven science, can offer better opportunities for healthcare providers to access real-time clinical information from patients and result in the collection of precision-health data that can better inform treatment strategies (Leth et al., 2017).

The development of high-precision wearable technologies and the myriad of sensors embedded within smartphones provide more detailed data that can be collected continuously over the duration of longitudinal follow-up (Malwade et al., 2018; Mahajan et al., 2020). Thus, transitioning to the digital space and deploying remote and continuous evaluations has the potential not only to improve feasibility but to overcome barriers to participation in research such as geographic location (participation of rural populations), low SES (individuals who cannot afford workdays off/transportation) and disadvantaged race/ethnic groups (segregated communities) (Canhoto and Arp, 2016; Malwade et al., 2018).

Advances in analytical techniques have also made it possible to analyze the wealth of multi-dimensional data collected digitally (Site et al., 2021; Sunny et al., 2022). Sensors in technology devices create additional data streams that provide greater insights into disease stages when used in conjunction with conventional clinical measures. Previous studies have developed digital health ecosystems to address gaps in mental health-care (Spadaro et al., 2021), diabetes management (Heintzman, 2015), and Parkinson’s disease symptom-monitoring and treatment (Ruokolainen et al., 2021). Among these, using machine learning models has shown great promise in predicting neurodegeneration in Parkinson’s disease and multiple sclerosis using digital technologies (Pratap et al., 2020; Zhang et al., 2020; Xue et al., 2021; Fröhlich et al., 2022).

Alzheimer’s disease (AD) and related disorders (ADRD) are well-suited for the implementation of digital-data collection ecosystems in clinical care and research settings. The heterogeneity of these conditions shows that there are multiple pathways by which an individual may develop disease. Etiological pathways tied to increased risk of ADRD include, but are not limited to, cardiovascular contributors (Stampfer, 2006), diabetes (Kroner, 2009; Michailidis et al., 2022), sleep disorders (Ju et al., 2013), and hearing problems (Lin et al., 2011; Liu et al., 2020). In fact, the recent Lancet Commission report indicated that across the life course, addressing a compilation of modifiable risk factors that, in combination, could reduce the risk for dementia/ADRD by as much as 40% (Livingston et al., 2020). The insidious nature of AD onset and progression is a natural setting in which to leverage digital health technologies that are sensitive enough to accurately detect the emergence of subtle clinical indicators (Kourtis et al., 2019; Sabbagh et al., 2020a).

Early diagnosis of AD has become the holy grail because of the presumption that treatment methods to date have been ineffective, given that interventions are too late and cannot reverse the pathological damage. The amyloid-tau-neurodegeneration (A/T/N) framework was developed to provide a diagnostic biomarker signature for the accurate diagnosis of AD at its earliest stage (Jack et al., 2016). However, currently accepted methods for detecting amyloid and tau are invasive and/or expensive and not even feasible in low-resource settings. For example, Positron Emission Tomography (PET) scans can cost as much as $10,000 per person per scan/tracer and require access to resources that are not scalable (Keppler and Conti, 2012; Al-Sharify et al., 2020). Cerebrospinal fluid (CSF) are currently the most accurate and least costly method for A/T/N verification, but participants frequently opt out because of the invasive nature of a spinal tap (Wojda, 2015). Moreover, neuropathological studies find that A/T/N biomarker positivity is not sufficient for a clinically expressed diagnosis (Carandini et al., 2019).

Neuropsychological (NP) testing to detect cognitive impairments is one of the most common methods for detecting clinically meaningful symptoms. However, NP testing requires training for standardized administration and scoring, placing a high burden on staff and individuals in clinical and research settings (Ruff, 2003). Many NP tests were developed and validated against a relatively homogenous population of European descent with relatively high education levels that is not applicable to the more general population, introducing bias and further increasing disparities in ADRD research (Loewenstein et al., 2008; Rivera Mindt et al., 2010; Fernandez, 2019). Thus, the utilization of remote digital technologies for NP testing provides the opportunity to reduce these barriers by providing access to highly specialized tools that can be operated at scale.

Despite the advantages of utilizing digital technologies in AD research and clinical care, there are concerns about uptake with older adults, since age remains the biggest risk factor for ADRD, and individuals with low technology literacy (Smith and Magnani, 2019). The prevailing mindset is that older adults will have difficulty or refuse to use digital technologies because they are unfamiliar with smart devices and unwilling to learn or get frustrated if the technology fails and stop participation, increasing the attrition rate of normal research/clinical use (Berenguer et al., 2017; Ware et al., 2017; Wild et al., 2019). Similarly, individuals with lower educational attainment tend and minoritized race/ethnic groups tend to have less engagement in technology health behaviors (Gordon and Hornbrook, 2016; Smith and Magnani, 2019). To address these concerns, it is essential to note that there are different classifications of technology that fall along the spectrum from active engagement to low/passive engagement.

Active engagement technologies require a participant to interact with the digital application/device, such as completing a cognitive task on a smartphone or syncing data on a wearable device (Lancaster et al., 2020; Sabbagh et al., 2020b; Au et al., 2021). Passive engagement technologies involve little to no interaction; these include sensor-based devices that are placed in the home, and can detect mobility, sleep, and breathing patterns, or in the car to capture driving behaviors (Kaye et al., 2012; Roe et al., 2017; Piau et al., 2019; Vahia et al., 2020; Au-Yeung et al., 2022; Wu et al., 2022). Passive engagement technologies also include smartphone applications that collect typing behaviors while participants use their phones without engaging in the application (Strickland, 2018; Lam et al., 2020). This is of high relevance given that mobile phones are the most penetrating device globally, with 73% uptake among individuals 10 or older across all countries and a 95% uptake in high income countries.^1^

Regardless of the instrument of digital data collection (e.g., smartphone, wearable, in-home device), it is important to rely on the same suite of sensors/functions from which to collect and interpret behavior (e.g., GPS, accelerometer, time-stamped coordinates, pressure, temperature, vibration, recorders, etc.). Thus, when using a suite of digital technologies, the same types of digital data streams are being collected from each of the instruments/applications. As long as the data collection protocol includes collection of digital data in its native format, it is possible to allow participants to pick different combinations of digital technologies that are best suited to their technology-use comfort level and still collect the data needed to measure the clinical outcomes of interest. Moreover, these assessments are low touch, meaning they can be self-administered, reducing staff burden or need for specific clinical training or expertise. This, in combination with the selection of assessment protocols that minimize biases (i.e., education, socioeconomic status, language, culture), makes it more feasible to implement at economies of scale, exponentially decreasing the financial and time burden posed by implementing fluid biomarkers or imaging modalities.

Here we present our approach to measuring cognitive and other AD-related behaviors that harness the power of a digital technology ecosystem using a participant-driven approach. The technologies we have chosen span into a variety of behavioral modalities, such as cognitively related measures assessed via smartphone applications, in addition to cardiovascular and sleep measures, physical activity, typing behavior, gait and balance, and voice recordings collected with wearable devices and mobile device-based sensors. This digital platform was developed with a device- and application-agnostic methodology to maximize the reach of the platform across different brain health-related measures. As innovative technologies emerge, we continuously search and test new digital devices to incorporate them into the platform, which serves to either expand our selection of digital technologies or replace existing ones. Furthermore, the deployment across cohorts with diverse participant characteristics from different sociodemographic and geographic settings allows for further tailoring of the digital platform and implementation processes making it possible to implement a “truly global” protocol that is more reflective of the world population.

## 2. Methods

We developed a custom platform with diverse off-the-shelf digital collection modalities to capture brain health-related measures. Digital data collection began in 2021 at both the Boston University Alzheimer’s Disease Research Center (BU ADRC) and the Bogalusa Heart Study (BHS). These two distinct and unique study populations provided an opportunity to assess how digital-health study design strategies can maximize inclusivity across high-resource and low-resource urban settings in the United States (US). Technologies and assessments were selected based on validation, usability, and access to data in its native format. Evaluation of the technology landscape through the lens of these criteria led to the curation of a multimodal platform accessible through both sites that captures cognition, speech, gait and balance, and questionnaires at both sites. The higher resource setting of the BU ADRC provided additional opportunities for extensive data collection, using multiple physical activity- and sleep-track wearables, active engagement smartphone and computer applications, and passive engagement motor activity and cognition monitoring applications. Prioritizing inclusivity has led to several protocol adjustments over the study period, ensuring that study activities suit the specific context of each cohort and the individual participant circumstances, which are central goals of the study.

### 2.1. Study populations

The BU ADRC and the BHS offer unique opportunities for scientific discovery on their own. Together they allow the exploration of disease-related processes across diverse demographic characteristics by leveraging existent clinical data. In addition, each site’s unique setting and geographic location allow refining a digital platform that is applicable and relevant to more diverse settings in the US, and that might be able to target emerging global-scale issues of ADRD.

#### 2.1.1. Boston University Alzheimer’s Disease Research Center

The BU ADRC is one of ~33 centers funded by the National Institute on Aging (NIA) that provide data to the National Alzheimer’s Coordinating Center (NACC) to promote collaborative research on AD. The study site is located in the urban area of Boston and includes community-dwelling older adults. Participants with and without cognitive impairment are longitudinally followed through annual neurological examinations, neuropsychological testing, clinical interviews, and additional procedures. A detailed description of the ADRC is provided elsewhere (Ashendorf et al., 2009; Gavett et al., 2012; Frank et al., 2022). A description of the NACC variables is available from the NACC website (National Alzheimer’s Coordinating Center, 2023). BU ADRC participants were invited to participate in our digital phenotyping project regardless of their cognitive status in order to include cognitively normal, mild cognitive impairment (MCI), or early AD individuals. All participants provided written informed consent, and Boston University Medical Center Institutional Review Board approved the digital protocol.

#### 2.1.2. Bogalusa Heart Study

The BHS is an ongoing, extensively characterized, population-based epidemiological cohort study that started in 1973. It has prospectively collected over five decades of repeated and longitudinal cardiovascular risk exposure data, in more than 1,000 individuals from childhood to midlife, with a high retention rate (Berenson, 2001). The substantial proportion of African Americans (35%), and the unique setting in the rural area of Bogalusa, Louisiana, have allowed thorough documentation of health disparities in cardiovascular risk over the life course (Freedman et al., 1988; Cruickshank et al., 2005; Wallace et al., 2013; Li et al., 2014). The BHS has collected midlife cognitive performance measures using traditional NP testing protocols as part of a previous examination (2013–2019), and are now entering their sixth decade of life. For the current study all participants provided written informed consent and the digital protocol was also approved by the Tulane Institutional Review Board.

### 2.2. Digital precision brain health monitoring platform

We developed a platform of digital technologies that collectively would produce brain-health related measures and multimodal digital data streams. This platform includes computer and smartphone applications and wearable devices and will continue to incorporate in-home sensors. A robust version of this platform is being used at the BU ADRC, whereas a subset of digital technologies has been introduced to BHS participants. The digital precision brain health monitoring platform includes nine available technologies for remote use and three for use in the research clinic. Technologies were identified and selected for inclusion in the digital phenotyping platform based on several criteria.

#### 2.2.1. Criteria for selection of technologies

Technologies were identified and selected based on several criteria including the validity of technology derived ADRD clinical measures, usability in older adults and individuals with low technological literacy, and access to raw digital data (e.g., in its native format) from the device or application. Operating system-agnostic technologies demonstrated the usability and existing validation for ADRD clinical measures.

#### 2.2.2. Validation and usability of technologies

The validity of different technologies was assessed and supported by previously published literature. We established an ongoing iterative evaluation of existing research across a range of lifestyle factors and clinical measures linked to AD (e.g., sleep, physical activity, smartphone-based cognitive assessment, gait and balance, cardiovascular health evaluation, etc.), that continues in parallel with prospective data collection. However, a great amount of the existent literature comes from other brain related disorders [e.g., traumatic brain injury, multiple sclerosis and Huntington’s disease (Lang et al., 2021)]. There is scarce evidence for the validity of digital measures in older adults with cognitive impairment, and several technologies with validation of measurement quality and feasibility were done on previous technology versions that have now been updated. Similarly, there was little evidence written in English that provided precedent of digital measures for ADRD in low-resource settings. Along with these drawbacks, research literature lacks the description of a digital protocol that is more inclusive, in which the context of use includes low-income, low technology literacy, and non-English speaking older adults with cognitive impairment for whom broadband access is limited or unavailable.

While we prioritized technologies that could distinguish between individuals with and without AD, in diverse populations and with large samples, no technology reported in the literature met all of these criteria. With the increasing investment in digital technologies and the rapid emergence of more advanced and improved applications and devices, relying on existing validation and usability against previously accepted non-digital standards will delay opportunities in determining the scientific value of digital technologies and derived metrics. We also prioritized technologies that are applicable across multiple operating systems. This study’s research protocol uses a “*bring your own device*” approach for the smartphone-based assessment. Digital tasks needed to work on both iOS and Android operating systems in order to increase inclusiveness in the study sample. Thus, our study will provide additional evidence for the validity and usability of digital technologies for ADRD used in a more diverse setting that is generalizable to more US population.

#### 2.2.3. Access to native format

Digital data in its raw native format was prioritized in technology selection in order to ensure longitudinal data integrity. Recognizing that hardware and software evolve rapidly and will continue to do so (Mathews et al., 2019), longitudinal digital collection protocols cannot be reliant on individual providers and their derived measures. Collecting raw digital data files enables comparisons across study participants who elect to use different technologies since the sensors collecting these data are similar across devices. Raw digital data files also ensure the validity of longitudinal data analysis as the platform changes because it allows more straightforward harmonization (Martinez-Murcia et al., 2020). Additionally, digital data in its native format allows for continuous updating of current features to meet contemporary algorithm standards and new feature generation from multiple types of analytics. The emphasis on raw digital data acquisition is carried throughout each platform component to enable adjustments to the study protocol while maintaining the opportunity for longitudinal analyses. Examples include the capture of phone sensor data (i.e., accelerometer, gyroscope, etc.) for the gait measurement and the derived measures of gait and balance. The digital voice capture protocol was developed to prioritize unstructured speech generation. Unstructured speech elicitation tasks ensure greater language and cultural adaptability of the assessment.

### 2.3. Selected technologies for multimodal data collection

The technologies in our precision brain health digital platform were selected to suit two different settings: urban higher income and semi-rural lower income. The strategy was to create a robust digital platform to maximize the digital footprint in both high internet-access environments and in areas where direct internet access is significantly more limited. The platform had to be flexible enough to be adapted to each study site and to individual contexts/preferences, and consistent enough to capture similar metrics across all participants. To accomplish this goal, a single multimodal smartphone-based assessment application, Linus Participant, was introduced as an assessment at both study sites.

**FIGURE 1:**
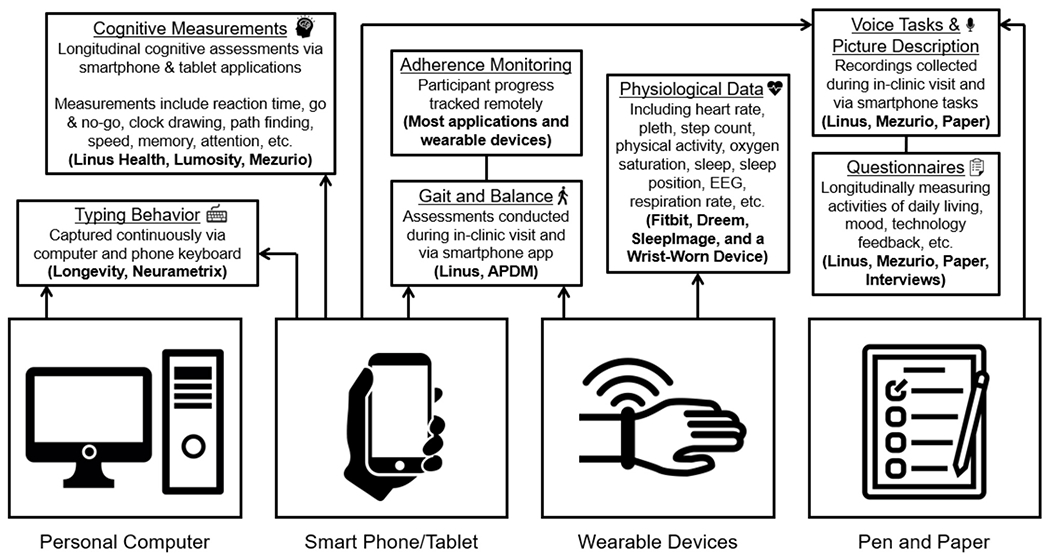
BU ADRC digital precision brain health platform data description.

At the BU ADRC, the nine remote engagement technologies included can be broadly classified into four groups: active engagement smartphone and computer applications, passive engagement smartphone and computer applications, wearables (physical activity and/or sleep), and staff-administered assessments (see Table 1). Five of the digital platform technologies were identified through literature and internet searches along the criteria previously described (digital data in native format, multiple OS, validity of derived metrics and usability). These technologies include a wrist-worn accelerometer, SleepImage ring, two smartphone applications (Lumosity and Linus Participant), and the NeuraMetrix typing cadence computer application. The other four remote technologies comprise the current Early Detection of Neurodegeneration (EDoN) initiative toolkit (Frey et al., 2019). The toolkit includes the Fitbit Charge 4 smartwatch, the Dreem3 electroencephalogram (EEG) headband, and two smartphone applications (Mezurio and Longevity). In-clinic technologies include the APDM Mobility Lab using Opal wearable sensors for gait and balance measurement, a tablet for digital pen data collection, and a handheld voice recorder (Frey et al., 2019). Staff-administered technologies include three iPad-based drawing tasks, a picture description and recall task using a digital voice recorder, and a gait task using wearable sensors. While several of the specific applications/devices included in the protocol are versions reported in the research literature (Tully et al., 2014; Thorey et al., 2020; Bezold et al., 2021; Zambelli et al., 2022), most of the technologies have since been updated, and the current versions have not been validated. The relatively long-time delay between data collection and publication of findings and frequent cycle of updates in the digital health technology landscape are a non-trivial concern in incorporating digital technology into longitudinal study designs. This conundrum reinforces the strategy of our digital platform design and further highlights the importance of collecting digital data in its raw native format, alongside any immediate derived measures of interest. Table 1 provides more details about the technologies employed in the digital protocol. Figure 1 offers an overview of the various types of data being collected via the platform.

### 2.4. Implementation of the digital technology platform

#### 2.4.1. Fitting the technology to the cohort

The utilization of a core multimodal smartphone application protocol, that is applicable across multiple sites, and the opportunities to expand based on the context and resources of each site parallels the approach at NACC. NACC has a Uniform Data Set (Kiselica et al., 2020) that is in use across the ADRC network, with multiple centers collecting similar data. Linus Participant includes digital assessments that cover different cognitive domains, processing speed, reaction time and executive function, as well as measurements of gait and balance, digital voice capture, and questionnaires. This application was selected because it only requires internet connection via a smartphone, is multimodal in its digital data types, and the time burden can be customized to the preference of the participant (e.g., shorter 5–10-min intervals, spread across multiple days or one 20–25 min test session). The speech elicitation tasks included in the platform have been adjusted toward more unstructured speech elicitation that are more culture, language and/or educational achievement agnostic (e.g., open-ended questions, picture description and recall, and semantic verbal fluency).

Participants at the BU ADRC site are given the option of selecting additional technologies from those listed in Table 1. All participants are presented with all available technologies that are in accordance with participant eligibility criteria (e.g., level of internet access).

At the Tulane BHS site, participants are recruited during in-clinic visits to the center that occur every 3–5 years. Study staff aids with the application’s setup and provides instructions for longitudinal use. The schedule for use is the same at the two sites. Additional technologies may be incorporated during the study period at the BHS site. Participants at both BU ADRC and BHS are asked to follow the customized digital protocol every 3 months across a 2-year period.

Over the initial 12 months of this project, a participant notification system was developed to ensure participants remained engaged with the study over time. At the BU ADRC, 3 weeks before each quarterly assessment period, participants are contacted to confirm availability at the planned time and to consider any changes to the selected personal technology schedule. This contact also offers an opportunity for troubleshooting with the participant. This is not implemented at the BHS as the logistics of adjustments, and shipping is unique to the BU ADRC protocol. At both sites, a manual notification is distributed at the start of the 2-week assessment period to remind participants to begin completing assessments. The Linus Participant app has also been configured to distribute notifications each day that an assessment is available for participants at both sites. At the BU ADRC, a second reminder is sent 1 week into the assessment to ask participants to complete all remaining assessments. Lastly, a third reminder is provided at the end of the 2-week assessment detailing what data has been collected from the participant. These reports offer an opportunity to address any issues, unexpected data points, or data quality concerns.

The methods and frequency of data uploads from technologies in the platform are provided in Table 1. Internet-connected devices and applications typically provide real-time or daily cadence updates through secure platforms or automated database queries. Derived measures accompany the reports for several technologies to identify possible data issues. Any updates on unresolved issues from previous assessments are also addressed before the start of the next assessment to ensure preparedness. Participants have the option of being contacted by email, text, or call based on their preference. Contact logs are maintained for each participant to allow for personalized communication.

#### 2.4.2. Personalizing the protocol to the individual

In addition to modifying the study protocol for the unique context and environment at each study site, protocol variations are implemented at the individual level to maximize inclusivity and longitudinal engagement. The personalization of the schedule is especially relevant to the BU ADRC site given the use of up to nine technologies and optional nature of technology selection.

Study staff describes how the technology is used, what measures are collected, and any benefits or risks of each device. The participant determines how many and which of the technologies they would like to use. A *preferred schedule of use* is offered, to use all technologies within 2-week assessment periods that occur every 3 months, but participants can adjust the schedule and period of use to meet their own preferences and need. The participant has the option to add and/or remove technologies throughout the study period. Participants are delivered a list of their selected technologies and affiliated manuals that include images to make instructions easier to comprehend and follow. Before their next 2-week window, participants provide qualitative feedback on the usability of each technology and any related burden developed. If the participant is able to travel for an in-clinic visit, they are asked to use the three in-clinic technologies during an annual 1 h in-clinic visit.

#### 2.4.3. Spectrum of usability and internet accessibility

Multiple devices were included across the key measurement domains of computerized cognitive assessment, passive digital phenotyping, physical activity measurement, cardiovascular health assessment, and sleep assessment in order to ensure the BU ADRC digital phenotyping platform is maximally flexible to participant preferences. Including multiple technologies that overlap in collecting similar measures help to ensure data for each measure of interest would be collected, despite individualized participant technology selections. Within each measurement domain, there are distinct characteristics which affect the participant experience. Many commercially available technologies include features that provide direct feedback to the user, such as step counts or sleep duration from a physical activity monitor or daily scores for brain games applications. These technologies are more scalable because they are developed for a commercial market, but these commercial devices might also be biased toward those with greater internet access (e.g., broadband in the home, greater smartphone data plans) and greater familiarity with digital technology. For example, wearable devices providing user-facing activity and sleep measures typically require a Bluetooth connection to a personal device, some frequency of charging, and may require daily engagement with an affiliated application. Our platform includes devices that appeal to participants with more or less internet access and/or low to high technology experience. There are several passive applications included that require no engagement from the participant after the initial set up. There are both wrist-worn devices that provide consumer grade information about steps and sleep duration and wrist-worn devices that record continuous digital data in its native format and can do so without any phone connection and without requiring any recharging of the battery. In this way, ease of use is balanced against providing informative information to the participant and inclusion of any participant, regardless of level of internet connectivity or technology familiarity.

#### 2.4.4. Recommended schedule and study flow

While both in-clinic and remote engagement opportunities were provided for participants, remote preference has become much more commonplace in the wake of the COVID-19 pandemic. Remote opportunities have facilitated additional engagement with individuals who have reduced mobility, who are outside of the Boston area, or who remain uncomfortable with extended in-clinic contact.

Parameters for use are provided to study participants. The parameters vary by technology according to the anticipated value of higher frequency collection as well as participant burden. Physical activity wearables in the protocol have a recommended schedule of 2 weeks of continuous wear. Sleep wearables have a recommended schedule of at least three nights of use during the two-week study period. Participants are asked to use the sleep devices at a lower frequency because of the higher burden to the participant in using the devices, coupled with participant feedback that more frequent use may disrupt sleep duration and quality. For active engagement smartphone applications, there were variations in frequency of self-administration. Active engagement schedules range from completion of five games for about 15 min total use over the 2-week compliance window to completion of 5–10 min of tasks each day for 2 weeks. The Linus Participant app in use at both study sites involved 25 min of tasks, which participants are asked to complete once over a 2-week timeframe.

### 2.5. Adjusting to real-world context

This study aims to take a pragmatic approach to observational research, with goals of maximizing inclusivity and opportunities for longitudinal engagement. Changes were made to the study protocol at multiple timepoints over the study period in line with these goals. It is possible that these changes may affect the analysis of the digital data collected, but each change was ultimately necessary to enable progress toward the goals of inclusivity and engagement. The inter-cohort and inter-participant heterogeneity offers exciting opportunities to address challenges that will continue to be present in digital collection protocols as they proceed to larger scale cohorts and real-world use.

The inclusion criteria at BU ADRC were modified to remove the requirement of smartphone ownership. These criteria excluded participants who had older phone models not considered to be “smartphones” or used another personal device, such as a tablet or a computer. This change was compatible with our study as several platform technologies were already accessible on a computer, tablet, or do not require any personal device.

Protocol modifications at the BU ADRC were instituted in efforts to ensure quality participant technology use while minimizing burden. Participant communication techniques were adjusted during the study period in line with participant preferences. Initially, only participants at the BU ADRC received notifications during their assessment period on account of the many technologies in use with the sample. This protocol was extended to the BHS site ~1 year after the initiation of data collection in order to improve adherence. The presence of staff contact has also been prioritized to provide clear opportunities for participants to notify study staff when technology glitches arise. In addition to notifications, data reports in the middle and at the end of 2-week assessment period were added for the BU ADRC site to provide feedback to participants and to provide an opportunity to readjust the protocol and schedule. The regular check-ins and weekly data reports support participants as the platform dashboard is tracking individual level participation.

Study activities have expanded over the course of the study period to include semi-formal interviews and general check-ins where participants can share their experiences with the technologies and any other aspect of the study. Individual assessments have been modified to account for ongoing evaluation of the possible biases included in testing prompts across multiple settings. Speech elicitation in the Linus Participant app has been adjusted at both study sites in order to replace structured prompts for unstructured prompts. Object recall, sentence reading, and story recall were replaced from the initial protocol for a picture description, picture description recall, and open-ended question tasks. Through avoiding tasks that ask participants to repeat provided stimuli, cultural biases in stimuli selection are now avoided.

Additional changes to the study recruitment criteria, study activities, and other aspects of the protocol will continue through the longitudinal follow-up period in line with the goals of maximizing inclusivity and longitudinal engagement.

## 3. Results

### 3.1. Recruitment and enrollment

Data collection was initiated in May 2021 at the BU ADRC and in September 2021 at the BHS. Through December 2022, 55 participants from the BU ADRC and 94 participants from the BHS have been recruited to participate in digital phenotyping. The demographic characteristics of the sample population at each site are provided in Table 2. The demographic characteristics of the study populations reflect the differences across study sites. The mean age in the digital phenotyping sample from the BU ADRC is 72.0 years-old, compared to 56.2 years-old at the BHS. The BU ADRC sample is 54.5% female and 10.9% Black, and the BHS sample is 69.8% female and 18.6% Black. There were also distinct educational level differences between cohorts, at the BU ADRC 75% had completed college and above, compared to 43.2% at the BHS sample. Cognitive diagnostic information is only available for the BU ADRC, and 20.0% of the sample has some diagnosed cognitive impairment.

### 3.2. Technology uptake

**FIGURE 2:**
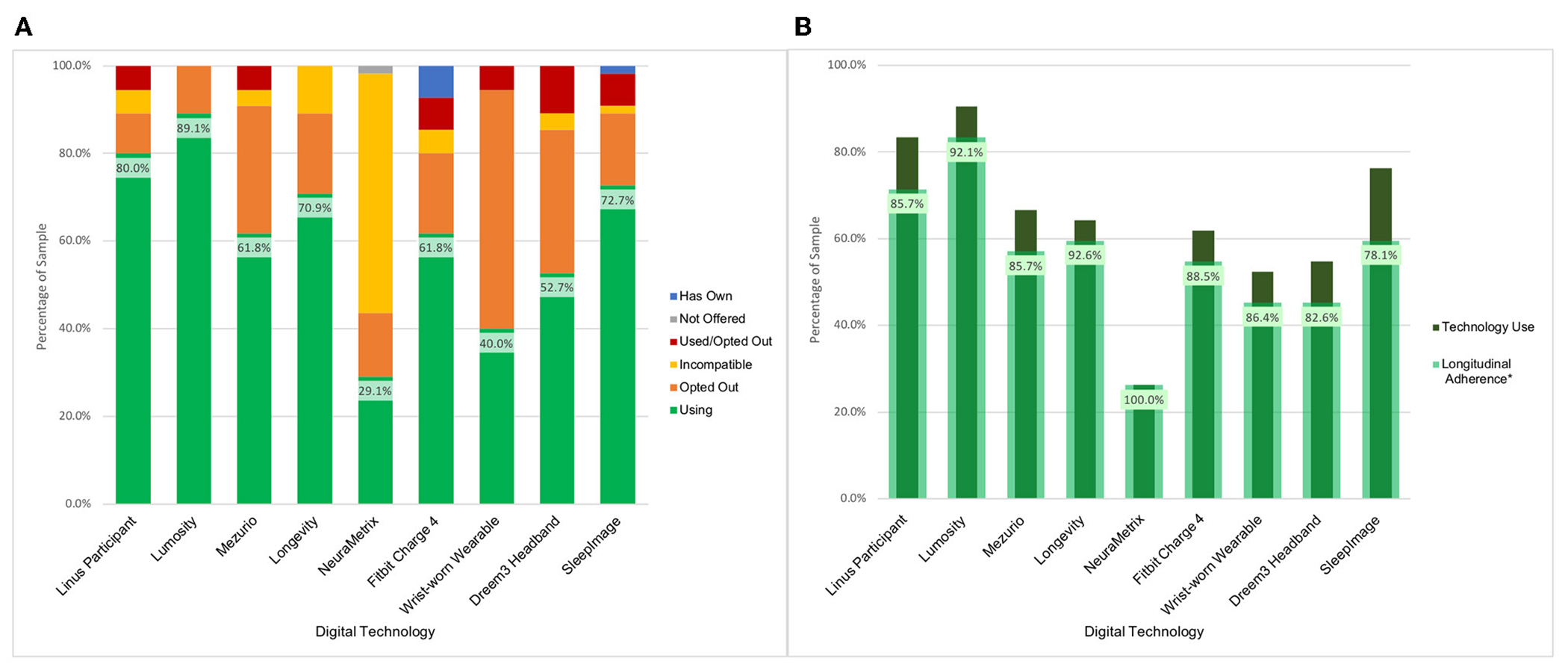
BU ADRC technology opt-in and longitudinal engagement description. **(A)** Description of participant technology selection. Definition of categories. Using: Participant is using the technology for prospective assessments as of December 2022. Not Offered: Technology was not presented to the participant as an option for selection as it was unavailable at time of enrollment. Used/Opted Out: Participant selected the technology and used it during an assessment period before opting to not use it for further assessments. Incompatible: Participant smartphone or computer characteristics prohibited use of technology. Opted Out: Participant was presented with the technology but opted not to use it. Has Own: Participant owns the technology and is not using it for study data collection. **(B)** Description of use of digital technologies among participants who have been followed across multiple measurement periods (*N* = 42). *Longitudinal adherence percent is presented as the percent of participants that have generated data at multiple check-in periods among those enrolled over multiple time points using a technology at any point.

BU ADRC participants have been offered nine technologies for remote use over their enrollment period. The majority of participants (*n* = 54, 98.2%) have expressed interest in using multiple technologies, with an average participant selection of 5.9 technologies. Uptake across the data collection modalities has been high which has resulted in a broadly phenotyped cohort. There was 87.3% (*n* = 48) uptake of at least one sleep device, 90.9% (*n* = 50) uptake of at least one physical activity device, and 98.2% (*n* = 54) uptake of at least one active engagement cognitive assessment application or web-based assessment. Across these technologies, 43 participants (78.2%) were engaged in parallel monitoring of sleep, activity, and active engagement applications measuring cognition (see Figure 2A).

BHS participants have been offered a smartphone-based application to date. Among the 94 participants who have opted into the study and have registered into the Linus App, 62 participants (68.1%) have completed at least one task using the Linus Participant smartphone-based platform. Among the 47 BU ADRC participants who have opted into the study and selected the Linus Participant app, 46 (97.9%) have completed at least one task.

#### 3.2.1. Technology withdrawals

The participant-driven technology selection has provided the opportunity to identify barriers to use, and what leads to technology acceptability. Based on opt-out decision patterns across technologies, the key factors that influenced the selection were the goals of the research project, the participant’s perceived value of the technology, and the device burden. The Dreem3 headband had the highest opt-out rate (60%) across initial technology selection and participant decision to opt-out after use (Figure 2A). Each technology included in the platform had at least a 10% opt-out rate.

Opt-out rates are provided as a proportion of person-technology combinations, a representation of each unique technology selected by each unique participant. This measure is reflective of how the characteristics of each technology (e.g., user feedback, charging requirement, time commitment) interact with characteristics of each participant (e.g., technology familiarity, work schedule, specific research contribution interest). There were 23 instances of a participant opting out of a device or application across 330 total person-technology combinations. The Dreem3 headband had the highest opt out rate of any technology with six opt-outs, and participants consistently attributed their decision to discomfort with the headband and/or effects on sleep. There were six total instances in which a participant decided to cease the use of a smartphone application, and all were driven by frustration with the functionality of the application or the completion of tasks. Technological glitches are an expected element in digital health research but can have significant effects on adherence and use. Overall, participants were more likely to opt-out of a wearable device after use than any of the smartphone applications. Of 188 total participant-app uses, six (3.2%) resulted in the participant deciding to opt out of the technology. Of 142 total participant-wearable uses, 17 (12.0%) resulted in the participant deciding to opt out of the technology. One factor contributing to participant opt-outs for wearable technologies was concern about the security of shipping devices back and forth due to participant’s living situations. At the BHS site, three participants opt-out of the Linus health platform. One participant stated that they no longer owned a smartphone, and the other two had no time to complete the assessments every 3 months.

#### 3.2.2. Technology ineligibility and issues

At the BU ADRC, the majority (58.1%) of participants had personal devices that would not allow the use of at least one technology. The NeuraMetrix computer typing cadence application had the highest percentage of ineligibility and the most stringent inclusion criteria; it can, at this stage, only be used by individuals with a Windows personal computer that only the participant used or through which the participant has their own Windows User Account. At the BHS site, 32 participants were unable to set up and register into the Linus Participant smartphone application at the time of enrollment due to internet connectivity issues. A set of instructions were provided via text messages and phone calls to complete the registration remotely. From these, a total of 14 (43.8%) participants have set up and started using the application remotely by following the instructions provided. Since sending the instructions, 18 (56.3%) participants remain unregistered, and these individuals are not included in the total of 94 participants who are engaged in the study. Since conducting the participant outreach, 14 (43.8%) of participants did not respond, 3 (9.4%) participants opted out of the study because of lack of time, and 1 (3.1%) opted out due to lack of cellular service.

Exclusion criteria vary across the technologies included in the platform. All devices have minimal operating system requirements, and all devices require available storage on participants’ personal devices. Operating system requirements resulted in one participant only being eligible for one application among the five which they had opted in for use.

#### 3.2.3. Longitudinal follow-up to date

Participants are scheduled to use the remote technologies for a 2-week assessment period every 3 months. As of December 2022, a total of 178 assessment periods have passed for 55 participants with a range of one to seven assessment periods per participant at the BU ADRC study site. Data has been collected in 171 (96.1%) of the assessment periods. Four (2.2%) assessment periods were skipped due to participant’s request (e.g., illness, planned vacation) and three (1.7%) were canceled due to no response. It is worth noting that data from passive technologies were still collected during these missed assessment periods. There have been 42 participants (76.4%) that have had at least two assessment periods during the study. Figure 2B indicates the Longitudinal Adherence Percentage for each technology—a measure describing the proportion of BU ADRC participants that have produced data at multiple time points for a given technology among those who have been enrolled for over 3 months. These figures demonstrate the variability in technology engagement over time. The technologies with the highest rate of use both in terms of participant selection and longitudinal follow-up are the smartphone and computer-based applications. Lumosity notably has the highest uptake and is the only technology that includes smartphone and computer interfaces, which expands the proportion of the sample which is eligible to include those with older operating systems.

There were 30 total instances of participants opting into a technology and not having at least two uses across the study period at the BU ADRC. The most common reason (*n* = 15, 50%) for a lack of longitudinal data is that a participant simply failed to complete their assessment. Missed assessments occur due to participant life events, such as loss of a family member or illness, as well as vacation, or simply forgetting to do a task and missing all notifications. Other causes for longitudinal data include opting out after a single use period (23.3%), technology issues preventing completion (6.7%), participants opting in after the beginning of the study period (16.7%), and participants purchasing their own device and ceasing study contributions (3.3%). The Longitudinal Adherence Percentage can be expected to increase over time for most of these cases, as participants have assessment periods remaining to re-engage with technologies.

At the BHS study site, 94 participants have been enrolled for more than 3 months as of December 2022. Among these individuals who have been asked to complete multiple self-guided assessments, 24 participants (38.1%) have completed assessments at multiple timepoints to date. Individuals that have not completed multiple assessments to date may still engage longitudinally. Longitudinal engagement is higher the longer participants are involved in the study. Among participants enrolled for five assessment periods, nine (47.4%) have completed tests at multiple timepoints. Among participants enrolled for four assessment periods, 16 (44.4%) have completed tests at multiple timepoints, and among participants enrolled for three assessment periods, 19 (41.3%) have engaged longitudinally.

## 4. Discussion

Use of the digital phenotyping platform in these two distinct observational cohorts demonstrates the feasibility of multimodal measurement. In the high-resource environment of the BU ADRC, high rates of uptake (>85%) across each modality of active engagement applications, sleep wearables, and activity wearables support the feasibility of robust digital data collection in older adult populations. With 78.2% of populations using at least one tool in each modality, the participant-driven pragmatic approach has demonstrated success in expanding the frequency and variety of data that can be collected from research participants. The BHS site has a comparatively lower rate of engagement with the multimodal smartphone app (68.1%), but the longitudinal use of the app also supports the feasibility of digital phenotyping in more semi-rural and low-resource environments. Changes were necessary throughout the study period to expand recruitment, engagement, and improve adherence. These lessons learned and the additional changes that will be needed for sustainable growth of these cohorts are described throughout the discussion.

### 4.1. Lessons learned

Successful enrollment and follow-up have relied on the use of a participant-driven approach. Digital phenotyping is a novel mechanism for data collection. Use of multimodal measurement for monitoring of brain health has required changes from the initial protocol that was established. Changes over the course of the study period include expanding participant eligibility to improve inclusivity, standardization of participant communication, schedule changes to reduce participant burden, and the expansion of the digital technology platform to include more applications and internet connected devices.

#### 4.1.1. Protocol design

The first challenge in pursuing this participant-driven scheduling with the BU ADRC was ensuring that proper IRB approval and consent language was in place. Introduction of off-the-shelf internet-connected digital health devices for human subject research will be a novel undertaking for many IRBs. Especially novel in this case is the introduction of over ten technologies within one study. Concerns from an institutional review perspective include the security of data stored by third-party vendors and the burden on study participants of multimodal engagement. Data privacy is addressed here through avoiding the sharing of identifiable data with technology vendors whenever possible through the use of coded IDs. In the case of technologies which may access identifiable data, institutional security reviews of vendor policies are required. Participant burden, in the BU ADRC study, is addressed through the optional nature of the digital technology platform.

#### 4.1.2. Recruitment

Recruitment into the digital phenotyping project has produced demographically distinct samples from the two study sites, with each providing opportunities for novel discovery. The BU ADRC sample has an average age of 72 years and includes participants with a diagnosed cognitive impairment. The age of the cohort provides further evidence of the feasibility of digital data collection in older adult populations, building off of the existing literature (Wilson et al., 2022). The BHS sample has a higher proportion of Black participants, a lower average age of 56 years, and is less-educated with only 43.2% of the sample with completed college. Through the successful recruitment of participants at the BHS site, the feasibility for the novel study design in semi-rural settings is supported.

Pragmatic evaluation of the study design produced the successful recruitment effort to date. Iterative protocol modifications described in the Section Adjusting to real-world context including expanding recruitment criteria, and utilizing a participant-centric design have supported efforts to build these digitally-phenotyped cohorts. These experiences demonstrate the importance of being device agnostic and considering back-dated operating systems in determining which technologies to include in digital phenotyping platforms. Discussion of participant burden candidly with prospective participants supported recruitment and follow-up also. Study staff highlighted several key points including that participation can be completely remotely for those who may not be comfortable or unable to come in-clinic.

#### 4.1.3. Reducing burden through tech use

Participant communication has been vital as is the case for any research study. Digital technologies offer opportunities for reducing burden through remote engagement, passive measurements, and self-administered testing. With participants at the BU ADRC using an average of 5.9 distinct remote technologies, burden remains a concern for this multimodal collection approach. Many participants at the BU ADRC typically did not engage with technology in their daily life, so feedback has been valuable to ensure the participant that their effort is leading to usable data.

Implementation of methods such as quarterly phone check-ins with participants and mid-assessment technology reports have helped to alleviate participant burden at the BU ADRC. Contact logs maintained for each participant and documentation of changes in technology use provide the most descriptive assessment of burden to date. Additional strategies for capturing participant burden through structured questionnaires are in development at both study sites, and a more detailed description of participant benefits and concerns are a future aim of this study.

Notably, the highest longitudinal engagement is demonstrated for passive applications that collect data in the background during participant’s typical use of their computer or smartphone. Passive monitoring is a promising tool for providing this opportunity for longitudinal engagement with minimal action needed from participants.

#### 4.1.4. Participant contact

The extent of participant communication is contingent on staffing, and has led to differing strategies for the two study sites. Manual reminders before, during, and after assessments for all participants are distributed for BU ADRC participants. At the BHS study, notifications are included during the assessment only for those who miss assessments in order to increase adherence. At the BHS study site, staff are responsible for multiple studies including recruitment and enrollment for another core clinical study. The BHS study also has a larger study sample, which makes individualized messaging less feasible. Participants at both sites are encouraged to contact study staff if they experience any issues with the technology.

Consistent communication procedures have improved participant engagement, information retention, and participant-staff interaction. The rate of longitudinal follow-up adherence is very likely attributable to the level of personal engagement. Empirical studies exploring the influence of personal contact as compared to automated engagement would be beneficial in further understanding the influence of personal contact on adherence (Killikelly et al., 2017; Lee et al., 2020).

#### 4.1.5. Monitoring and adherence

Results regarding participant’s longitudinal engagement, reasons for opting out, and reasons for non-adherence would not be available without frequent adherence evaluations conducted by staff. The ability to collect and upload data in real-time is an important factor for consideration when selecting technologies for research or clinical use. Without real-time (or near real-time) data flows, tracking adherence and data quality may only be accessible after data loss has occurred. Detailed documentation for derived estimates of adherence or data quality is also an important factor for consideration, as bias in these estimates could result in inaccuracies in generated data. It may also be the case that inability to use technology appropriately or according to a defined schedule may be indicative of significant cognitive impairment (Sanborn et al., 2019). Therefore, initial adherence followed by reduced adherence over time may be data, apart from any data collected from the digital application/device. Additional research on non-adherence and error rates in technology use as an indicator of cognitive impairment and pathological neurodegeneration are needed. Failure to remember the schedule for self-guided assessments or confusion over application instructions could also be indicative of changes in cognition. Focusing solely on digital data capture without tracking approaches to increase adherence may be a missed opportunity for detecting an important behavioral change.

#### 4.1.6. Smart automation

A key benefit of digital monitoring is the opportunity to conduct studies at a larger scale with less resources devoted to study staff and infrastructure. The responsibilities associated with study upkeep have grown steadily as participation has increased. The study team utilizes a project management tool to automate and track different aspects of the study including recruitment, participant adherence, shipping and receiving, and troubleshooting. Smart automations remind staff and generate tasks for when participants have an upcoming assessment period or need a technology adherence report. This infrastructure supports the study staff on a day-to-day basis and maintains consistency across all participants despite the variability in technology selection and communication preferences. Similar management systems may not be practicable or accessible in all research or clinical environments. Moving forward, an additional factor in technology selection will be the capacity of technologies to provide effective participant adherence monitoring and support without staff oversight. Most technologies have some automatic reminder system, but the contrast in adherence between the BU ADRC and BHS sites demonstrates the difference in effectiveness for universal reminders (such as the daily reminders from the Linus Participant app) compared to personalized check-ins and reminders (such as BU ADRC weekly data reports). Further research is needed to explore what features of participant notifications can drive participant engagement. Some variations identified across the BU ADRC digital technology program include personalized timing of notifications, modifiable text for notifications, and notification method (e.g., phone call, text message, email, app push notification). Challenges in maintaining standardized and sufficient contact with study participants provide support for the importance of passive monitoring technologies. Passive engagement applications require minimal effort from staff and participants. The current major caveat is greater concerns over personal privacy and data security. At the BU ADRC, interestingly, uptake rates for passive monitoring technologies have been influenced by technology incompatibility rather than concerns for security.

### 4.2. Next steps

#### 4.2.1. Characteristics of an ideal technology

Experiences with platform technologies to date will inform the identification of new technologies to implement across the study sites. Moving forward, we plan to prioritize passive data collection technologies and smartphone applications for inclusion in the platform. Technology uptake rates and longitudinal use demonstrate the greater uptake of applications as compared to wearable devices. Applications are also more scalable to low resource settings where the cost of additional hardware for wearable technologies may not be feasible. Passive data collection will be prioritized as it can be conducted with minimal engagement, but privacy and increased data security issues need to be addressed in tandem. All platform technologies need to work across different operating systems.

#### 4.2.2. Data management and sharing

The multimodal nature of the platform and the participant-driven technology selection will require advanced analytic tools for reliable analyses. The heterogeneity of techniques used by participants poses a challenge to the comprehensive utilization of multimodal information using traditional analysis methods (Mohr et al., 2013). Novel procedures will need to be developed for the capturing, storing, cleaning, processing, analyzing, and sharing of both derived digital metrics and the raw digital data streams (Mohr et al., 2013). Future plans include processing all data and sharing data through various interoperability platforms, such as the Alzheimer’s Disease Data Initiative (ADDI). Collaborative efforts with academic and industry partners, as well as citizen scientists will be needed to capitalize on global analytic expertise. Through providing a range of complex, real-world data streams, the BU ADRC and BHS digital programs can enable creation of processing and quality control systems that are applicable across a range of technologies. The development of these systems within a pre-competitive academic center will facilitate widespread use.

Current efforts are underway to share the digital data in its native format through ADDI (2022), but do so in a way to maximally protect privacy and confidentiality. The digital data will be accompanied with documentation and tools for processing across the data management and analysis pipelines. Providing raw data through the ADDI and other data sharing platforms will also enable the global research community with opportunities to develop and test their own processing pipelines and extract information from collected data that no single team may consider. The various technologies included in the platform will likely provide data that is useful beyond the scope of this project. To extend the utility of the digital data collection, an additional element of the research protocol is the development of consent language that permissions longitudinal use of digital data for use beyond those anticipated at the time of participant enrollment.

#### 4.2.3. Fitting the analysis to the method

Traditional biostatistics analytic approaches are not well-suited to fully capture the rich, but heterogenous digital data captured through this participant-driven protocol. Inconsistency in the selection of technologies will limit the sample size for evaluating associations between measures from specific sensors and clinical outcomes. Analyzing the full scope of the digital phenotypes captured by the different sensors (gait, reaction time, voice, etc.) will require substantial efforts in novel harmonization in research data. Our efforts will seek to expand upon recent work in harmonization of intensive longitudinal data (Chow et al., 2023). In addition to providing a rich data resource, this project also aims to provide a system through which digital data could be collected, processed, and later analyzed at scale.

## 5. Conclusion

Longitudinal engagement with multimodal digital health applications in demographically heterogenous samples supports the hypothesis that digital technologies can act as a tool for further equity in research. Utilization of a pragmatic, cohort-adapted, and participant-driven study design enabled engagement with digital collection via a multitude of digital sensors at the BU ADRC and the BHS. Ongoing data collection offers opportunities for refining digital technology selection criteria and digital phenotyping protocol development. Collected data will be instrumental in the development of novel systems for the processing, storage, QC, and future analysis of multimodal digital data streams.

## Figures and Tables

**TABLE 1:** Digital brain health monitoring platform: technology use and data description.

	Technology	Platform	Measures	Participant schedule	Assessment frequency	Compliance tracking	Data upload
Active engagement application	Linus participant	Smartphone	Cognition, speech, gait and balance, questionnaires	Complete 25min series of tasks once remotely	Quarterly	Daily	Automatic upload (w Internet)
	Lumosity	Smartphone, computer	Cognition	Complete 5 tasks (15min) remotely	Quarterly	Bi-weekly	Automatic upload (w Internet)
	Mezurio	Smartphone	Cognition, speech, motor function	Complete 10min of tasks daily for 2 weeks remotely	Quarterly	Daily	Automatic upload (w Internet)
Passive engagement application	Longevity	Smartphone	Motor function	Data collected continuously in the background	Continuous^x^	Weekly	Automatic upload (w Internet)
	NeuraMetrix typing cadence application	Computer (PC Only)	Cognition, motor function	Data collected continuously in the background	Continuous^x^	Daily	Automatic upload (w Internet)
Activity wearable	Fitbit charge 4	Wrist-worn device, smartphone	Physical activity, heart rate, sleep	Wear wrist device day and night for 2 weeks	Quarterly	Daily	Automatic upload (w Internet and Bluetooth)
	Wrist-worn wearable	Wrist-worn device	Physical activity, sleep	Wear wrist device day and night for 2 weeks	Quarterly	One time upload^**^	Manual upload by staff
Sleep wearable	Dreem3 headband	Head-worn device, Smartphone	Sleep movement, respiration, EEG	Wear sleep headband for 3 or more nights	Quarterly	Daily	Automatic upload (w Internet) when charging
	SleepImage	Finger-worn device, Smartphone, cloud-computing	ANS-based sleep evaluation, pulse oximetry, respiration, sleep	Wear sleep ring for 3 or more nights	Quarterly	Daily	Automatic Upload (w Internet and Bluetooth)
Staff-guided technology	APDM mobility lab*	Wearable sensor system	Motion, gait and balance metrics	Complete set series of tasks in person	Annually	Not applicable	Staff guided after assessment
	Linus tester*	Tablet	Digital pen movement, multi-domain cognition	Complete set series of tasks in person	Annually	Not applicable	Staff guided after assessment
	Digital voice recorder*	Voice recorder	Voice	Complete picture description and recall in person or remotely	Annually	Not applicable	Staff guided after assessment

* Device used during staff-guided assessments. Gait and balance sensors and tablet-based assessment are used only during in-clinic visits. Digital voice recorder is used during staff-guided in-clinic and remote visits.

** The wristband continuously collects data from an embedded accelerometer after study staff links the device to a study-based smartphone. Then, the collected data is uploaded manually to an Amazon Web Services (AWS) backend when the technology is returned to the study site.

x Continuous smartphone and computer tracking entails the capture of typing speed and usage data whenever a participant uses their personal device.

**TABLE 2:** Demographic characteristics of study populations.

Variable	BU ADRC* (*N* = 55)	BHS (*N* = 94)
**Age**		
Mean (SD)	72.0 (8.0)	56.2 (4.5)
**Sex**		
Female	30 (54.5%)	60 (69.8%)
Male	22 (40.0%)	26 (30.23%)
**Race**		
Black or African American	6 (10.9%)	16 (18.6%)
White	46 (83.6%)	70 (81.4%)
**Education**		
Less than high school degree (<12 years ed)	–	4 (3.76%)
Less than college degree (12–15 years ed)	13 (25.0%)	44 (41.36%)
College degree and above (16+ years ed)	39 (75%)	46 (43.24%)
**Cognitive status**		
Cognitive normal	41 (74.5%)	NA
Cognitively Impaired	11 (20.0%)	NA

* Information for the BU ADRC sample is still being compiled. Demographic information for 52 participants is provided.

NA, Not applicable. Participants are relatively young and come from an observational cohort study of community-dwelling adults with no clinical diagnosis of cognitive impairment.

## Data Availability

The raw data supporting the conclusions of this article will be made available by the authors, without undue reservation.

## Footnotes

1 *Facts and Figures 2022: Mobile phone ownership. | International Telecommunication Union*. https://www.itu.int/itu-d/reports/statistics/2022/11/24/ff22-mobile-phone-ownership/.
